# Polymorphism in the Serotonin Receptor 2a (*HTR2A*) Gene as Possible Predisposal Factor for Aggressive Traits

**DOI:** 10.1371/journal.pone.0117792

**Published:** 2015-02-06

**Authors:** Zsofia Banlaki, Zsuzsanna Elek, Tibor Nanasi, Anna Szekely, Zsofia Nemoda, Maria Sasvari-Szekely, Zsolt Ronai

**Affiliations:** 1 Department of Medical Chemistry, Molecular Biology and Pathobiochemistry, Semmelweis University, Budapest, Hungary; 2 Institute of Psychology, Eotvos Lorand University, Budapest, Hungary; University of Medicine & Dentistry of NJ—New Jersey Medical School, UNITED STATES

## Abstract

Aggressive manifestations and their consequences are a major issue of mankind, highlighting the need for understanding the contributory factors. Still, aggression-related genetic analyses have so far mainly been conducted on small population subsets such as individuals suffering from a certain psychiatric disorder or a narrow-range age cohort, but no data on the general population is yet available. In the present study, our aim was to identify polymorphisms in genes affecting neurobiological processes that might explain some of the inter-individual variation between aggression levels in the non-clinical Caucasian adult population. 55 single nucleotide polymorphisms (SNP) were simultaneously determined in 887 subjects who also filled out the self-report Buss-Perry Aggression Questionnaire (BPAQ). Single marker association analyses between genotypes and aggression scores indicated a significant role of rs7322347 located in the *HTR2A* gene encoding serotonin receptor 2a following Bonferroni correction for multiple testing (p = 0.0007) both for males and females. Taking the four BPAQ subscales individually, scores for Hostility, Anger and Physical Aggression showed significant association with rs7322347 T allele in themselves, while no association was found with Verbal Aggression. Of the subscales, relationship with rs7322347 was strongest in the case of Hostility, where statistical significance virtually equaled that observed with the whole BPAQ. In conclusion, this is the first study to our knowledge analyzing SNPs in a wide variety of genes in terms of aggression in a large sample-size non-clinical adult population, also describing a novel candidate polymorphism as predisposal to aggressive traits.

## Introduction

Aggression, defined as any behavior intended to be destructive, lies at the root of numerous major ills of humanity ranging from verbal abuse through both interpersonal and self-directed violence to mass criminal acts. Consequences of aggression-driven acts pose an enormous burden on society and economics, rendering it important to understand the biological basis behind [1,2].

Increased levels of aggression are characteristic to patients with a variety of neurodegenerative and psychiatric disorders as well as to alcoholics and drug addicts [3–7], but can also often be observed among the normal human population, even conferring certain privileges to the aggressor under certain circumstances e.g. by means of social dominance [8,9]. From the evolutionary point of view, some degree of aggression is indeed necessary for gaining adequate fitness (through an improved access of food supplies and other resources) and reproductive success; however, these benefits are compensated for by an increased risk of injury and social isolation. Hence, optimal levels of aggression are presumably shaped by a fine balance between effects of positive and negative selection pressure, implying a strong genetic background next to the role of environment [10,11]. This assumption is further underpinned by the fact that aggression proved to be heritable in several twin studies, with an estimated genetic contribution to the risk of aggressiveness of above 40% [12–17].

Experimental evidence suggest that aggressive manifestations and the accompanying emotions (anger, anxiety, fear) can be strongly related to highly conserved brain regions, chiefly to the amygdala and its linked neural circuits, but also to the anterior cingulated cortex and the prefrontal cortex [18,19]. In terms of biochemistry, it is principally the monoaminergic neurotransmitter systems (e.g. dopamine, noradrenaline and serotonin pathways) that are believed to play a major role in aggressive behavior, though possible effects of sexual hormones, the hypothalamic-pituitary-adrenal (HPA) axis and blood sugar levels have also been implicated [20,21].

Great efforts have been made to decipher the possible genetic background behind predisposition to aggression, describing novel polymorphisms in a variety of genes with a role in neuropsychiatry, and also identifying promising candidates for aggressive behavior and the related mental states (impulsivity, hostility). However, most of these association studies were carried out in small samples, raising the possibility of committing statistical errors (Pavlov 2012). Besides, the vast majority of aggression-related genetic investigations either were based on comparisons between healthy individuals and patients suffering from personality disorders etc., or concentrated on restricted samples not representative of the general population (e.g. [22–28]). These factors render data evaluation challenging, and often lead to controversial results.

Our aim was to simultaneously examine the effect of a set of putatively functional single nucleotide polymorphisms (SNP) on aggressive tendencies of the general Hungarian adult population using a microarray system, with a principal focus on monoaminergic pathways and its close interactors. Selected SNPs are located in genes encoding monoaminergic neurotransmitter transporters and receptors, their associated proteins and other signal transduction molecules, enzymes involved in the biosynthesis or degradation of neurotransmitters, neurotrophic factors and regulators of circadian rhythm as well as of neuronal death, all with an implicated role in emotional responses and behavioral traits [20,29–32].

## Materials and Methods

### Individuals involved

Non-related individuals of Caucasian Hungarian origin without any known psychiatric disorder were recruited for this study on a voluntary basis at the Institute of Psychology, Eotvos Lorand University (Budapest). Buccal samples and self-filled out aggression questionnaires were obtained from 887 subjects (45.8% males and 54.2% females). The sample comprised of 495 psychology and law enforcement students studying in the Budapest area and 392 random volunteers recruited at academic institutions and events popularizing this survey. All participants belonged to the middle socioeconomic status. Mean age was 23.2 (±7.55) years within the range from 18 to 75 years. All participants gave written informed consent and the study was approved by the Scientific and Research Ethics Committee of the Medical Research Council (“ETT TUKEB”—Ministry of Health, Medical Research Council, Budapest, H-1051 Hungary).

### Phenotypic measure

The original 29-item version of the self-report Buss-Perry Aggression Questionnaire (BPAQ) [33] was used to assess aggressive tendencies. This instrument comprises four subscales: Verbal Aggression (5 items), Physical Aggression (9 items), Anger (7 items) and Hostility (8 items). Individual items are rated from one (‘extremely uncharacteristic of me’) to five (‘extremely characteristic of me’). Total score for aggression was calculated as the sum of ratings for all the items, with a possible range between 29 and 145. Hungarian version of the original English language questionnaire was obtained by the “forward-backward” translation method and was pilot tested prior to the present study [34].

### Sample collection

Buccal cells were collected by gently scraping the inner cheek with cotton-tipped collection swabs. Genomic DNA preparation was performed by a traditional, salting-out procedure [35]. Briefly, collection swabs were incubated overnight in 450 μl cell lysis buffer (0.2 g/l Proteinase K, 0.1 M NaCl, 0.5% SDS, 0.01 M Tris buffer pH = 8.0) at 56°C, followed by RNase treatment at room temperature. Proteins were precipitated with saturated NaCl (6 M) and removed by centrifugation. DNA was precipitated with isopropanol, purified with 70% ethanol and resuspended in 100 μl of Tris-EDTA pH = 8.0 (containing 0.5 M EDTA). DNA concentrations were measured by a fluorometry based intercalation assay (AccuBlue Broad Range dsDNA Quantification Kit, Biotium). Concentration of samples analyzed in this study ranged between 15 and 200 ng/μl. Isolated DNA samples were kept at −20°C until used.

### Marker selection

Common SNPs with a higher than 5% minor allele frequency (MAF) were selected from the dbSNP database of NCBI [36]. Priority was given to polymorphisms referred to in various association studies in connection with personality or mood disorders as well as aggression or impulsivity in psychiatric disorders, and to putative functional variants, either causing an amino acid change or with an implicated gene regulatory role.

### Genotyping

Genotyping was performed in 384-well plates on an Open Array real-time PCR platform (Applied Biosystems) based on allele-specific, fluorescent (TaqMan) probes and pre-designed, validated primers immobilized to a solid surface obtained from the manufacturer. Approximately 100 ng DNA per sample was used in each measurement. DNA amplification was carried out in the GeneAmp PCR System 9700 (Applied Biosystems) according to the manufacturer’s instructions, using the master mix, containing each dNTP and AmpliTaq Gold DNA-polymerase, provided by the manufacturer. Endpoint detection of signal intensities of allele specific fluorescent dyes was conducted by the OpenArray NT Imager, and genotypes were called by the TaqMan Genotyper v1.2 software. Call rate for individual SNPs is shown in Table 1 (mean: 77.9%).

**Table 1:** Genotype distribution of the studied SNPs.

	SNP	Gene	N	Genotype						HWE*	Call rate
				MM		Mm		mm			
1.	rs1048101	*ADRA1A*	763	218	28,6%	384	50,3%	161	21,1%	0.945	86%
2.	rs3808585	*ADRA1A*	722	396	54,8%	277	38,4%	49	6,8%	0.998	81%
3.	rs2236554	*ADRA1D*	757	293	38,7%	346	45,7%	118	15,6%	0.641	85%
4.	rs553668	*ADRA2A*	692	519	75,0%	158	22,8%	15	2,2%	0.770	78%
5.	rs11030104	*BDNF*	702	393	56,0%	264	37,6%	45	6,4%	0.997	79%
6.	rs2049045	*BDNF*	690	419	60,7%	241	34,9%	30	4,3%	0.820	78%
7.	rs6265	*BDNF*	601	362	60,2%	212	35,3%	27	4,5%	0.847	68%
8.	rs7103411	*BDNF*	715	393	55,0%	276	38,6%	46	6,4%	0.966	81%
9.	rs7094179	*CDNF*	687	305	44,4%	302	44,0%	80	11,6%	0.924	77%
10.	rs7900873	*CDNF*	696	384	55,2%	273	39,2%	39	5,6%	0.573	78%
11.	rs1051730	*CHRNA3*	753	320	42,5%	345	45,8%	88	11,7%	0.943	85%
12.	rs16969968	*CHRNA5*	663	279	42,1%	307	46,3%	77	11,6%	0.866	75%
13.	rs4680	*COMT*	603	177	29,4%	295	48,9%	131	21,7%	0.927	68%
14.	rs135745	*CSNK1E*	718	187	26,0%	375	52,2%	156	21,7%	0.460	81%
15.	rs1997644	*CSNK1E*	688	176	25,6%	364	52,9%	148	21,5%	0.291	78%
16.	rs1611115	*DBH*	761	443	58,2%	283	37,2%	35	4,6%	0.482	86%
17.	rs6271	*DBH*	780	657	84,2%	116	14,9%	7	0,9%	0.759	88%
18.	rs4532	*DRD1*	761	286	37,6%	357	46,9%	118	15,5%	0.931	86%
19.	rs6277	*DRD2*	579	169	29,2%	284	49,1%	126	21,8%	0.948	65%
20.	rs1800497	*DRD2*	605	399	66,0%	192	31,7%	14	2,3%	0.261	68%
21.	rs1079597	*DRD2*	608	443	72,9%	158	26,0%	7	1,2%	0.226	69%
22.	rs1800498	*DRD2*	595	215	36,1%	280	47,1%	100	16,8%	0.862	67%
23.	rs2134655	*DRD3*	760	410	53,9%	295	38,8%	55	7,2%	0.981	86%
24.	rs3732790	*DRD3*	734	243	33,1%	365	49,7%	126	17,2%	0.857	83%
25.	rs6280	*DRD3*	749	354	47,3%	326	43,5%	69	9,2%	0.887	84%
26.	rs963468	*DRD3*	736	246	33,4%	364	49,5%	126	17,1%	0.909	83%
27.	rs11246226	*DRD4*	685	173	25,3%	347	50,7%	165	24,1%	0.941	77%
28.	rs3758653	*DRD4*	714	486	68,1%	208	29,1%	20	2,8%	0.923	80%
29.	rs916455	*DRD4*	702	644	91,7%	56	8,0%	2	0,3%	0.803	79%
30.	rs936460	*DRD4*	697	344	49,4%	284	40,7%	69	9,9%	0.655	79%
31.	rs3733829	*EGLN2*	683	263	38,5%	321	47,0%	99	14,5%	0.998	77%
32.	rs222843	*GABARAP*	683	307	44,9%	293	42,9%	83	12,2%	0.601	77%
33.	rs11111	*GDNF*	719	540	75,1%	160	22,3%	19	2,6%	0.241	81%
34.	rs1549250	*GDNF*	710	231	32,5%	353	49,7%	126	17,7%	0.907	80%
35.	rs1981844	*GDNF*	576	320	55,6%	223	38,7%	33	5,7%	0.771	65%
36.	rs2910702	*GDNF*	705	387	54,9%	269	38,2%	49	7,0%	0.971	79%
37.	rs2973041	*GDNF*	695	492	70,8%	182	26,2%	21	3,0%	0.710	78%
38.	rs2973050	*GDNF*	582	242	41,6%	275	47,3%	65	11,2%	0.608	66%
39.	rs3096140	*GDNF*	671	320	47,7%	287	42,8%	64	9,5%	1.000	76%
40.	rs3812047	*GDNF*	679	521	76,7%	144	21,2%	14	2,1%	0.559	77%
41.	rs6925	*HTR1A*	607	167	27,5%	289	47,6%	151	24,9%	0.510	68%
42.	rs1228814	*HTR1B*	599	432	72,1%	153	25,5%	14	2,3%	0.995	68%
43.	rs130058	*HTR1B*	595	330	55,5%	232	39,0%	33	5,5%	0.642	67%
44.	rs13212041	*HTR1B*	606	376	62,0%	209	34,5%	21	3,5%	0.467	68%
45.	rs11568817	*HTR1B*	600	187	31,2%	292	48,7%	121	20,2%	0.937	68%
46.	rs6296	*HTR1B*	607	325	53,5%	233	38,4%	49	8,1%	0.730	68%
47.	rs6311	*HTR2A*	777	243	31,3%	391	50,3%	143	18,4%	0.809	88%
48.	rs6313	*HTR2A*	769	240	31,2%	385	50,1%	144	18,7%	0.893	87%
49.	rs6314	*HTR2A*	773	640	82,8%	130	16,8%	3	0,4%	0.409	87%
50.	rs7322347	*HTR2A*	765	242	31,6%	370	48,4%	153	20,0%	0.866	86%
51.	rs7984966	*HTR2A*	758	411	54,2%	293	38,7%	54	7,1%	0.984	85%
52.	rs3813929	*HTR2C*	744	555	74,6%	117	15,7%	72	9,7%	0.975	84%
53.	rs518147	*HTR2C*	717	379	52,9%	166	23,2%	172	24,0%	0.237	81%
54.	rs6318	*HTR2C*	769	570	74,1%	127	16,5%	72	9,4%	0.737	87%
55.	rs907094	*PPP1R1B*	705	409	58,0%	246	34,9%	50	7,1%	0.308	79%

M: major allele, m: minor allele

*Hardy Weinberg Equilibrium.

### Statistical analysis

Statistical analyses were performed by the SPSS 22.0 (SPSS Inc.) software. Allele and genotype frequency distributions were determined by the *χ* ^2^ test. Independent samples t-test was used to assess gender differences, and relationship with age was tested by Pearson correlation. Genetic associations were tested by one way analysis of covariance (ANCOVA) assuming a dominant model of inheritance with sex and age as covariates. Bonferroni correction for multiple testing was applied for the total number of SNPs in this study when assessing relationship between BPAQ scores and individual SNPs (the corrected level of significance was p = 0.05 / 55 = 0.0009). In all other cases, p ˂ 0.05 values were regarded as significant. Effect of prior associations in males and females was analyzed by two-way ANCOVA with age as covariate. All tests were two-tailed. Lewontin’s *D’* and *r* ^2^ values of linkage disequilibrium were calculated using HaploView 4.2. [37]. Haplotypes were determined by the PHASE software [38,39].

## Results

### Reliability of the markers analyzed

Internal consistency of the self-report BPAQ was assessed by Chronbach’s alpha, which had a value of 0.895 for total scores ensuring reliability of the study. Coefficients for Verbal Aggression, Physical Aggression, Anger and Hostility were 0.640, 0.842, 0.831 and 0.792, respectively.

Alleles of all the SNPs studied were in Hardy-Weinberg equilibrium (Table 1).

### Potential confounders

Gender differences on the BPAQ scale were evaluated by Independent samples t-test. Males presented significantly higher scores than females (68.52±17.14 compared to 64.49±15.09; p<0.001). Relationship between BPAQ scores and age was tested by Pearson correlation coefficient and was found to be significant (p = 0.008). Thus, both gender and age were used as covariates in all association analyses.

### Significant association of the HTR2A rs7322347 T/A intronic SNP with aggression

Table 2 summarizes results of phenotypic data as a function of each SNP analyzed. Association with aggression reached nominal level of significance p<0.05 in the case of two SNPs, rs916455 located in the promoter region of the *DRD4* gene and rs7322347 in intron 2 of *HTR2A*. Corresponding statistical values for these were [F = 4.878, p = 0.0275, η^2^ = 0.007, power = 0.597] and [F = 11.617, p = 0.0007 η^2^ = 0.015, power = 0.926], respectively. In order to reduce the likelihood of a type I error, Bonferroni adjustment on the target alpha level was performed to correct for multiple testing. Effect of the rs7322347 polymorphism remained significant after Bonferroni-correction, labeled by an asterisk in Table 2. Individuals homozygous for the wild type allele (T) of rs7322347 had significantly higher aggression scores (69.21±17.00) compared to those carrying at least one minor allele (A) of this polymorphism (65.34±15.69). The corresponding Cohen’s d effect size for rs7322347 was d = 0.24.

**Table 2:** Association of the 55 polymorphisms studied with aggression levels.

	SNP	Gene	Aggression (total score)			p^#^
			MM	Mm	mm	
1.	rs1048101	*ADRA1A*	66.66	66.50	66.46	0.9684
2.	rs3808585	*ADRA1A*	66.15	68.19	65.93	0.2294
3.	rs2236554	*ADRA1D*	65.31	67.18	68.29	0.0840
4.	rs553668	*ADRA2A*	66.52	66.61	70.47	0.8682
5.	rs11030104	*BDNF*	66.56	66.60	67.73	0.8735
6.	rs2049045	*BDNF*	66.34	67.15	66.73	0.5703
7.	rs6265	*BDNF*	66.94	66.80	65.98	0.9220
8.	rs7103411	*BDNF*	66.55	66.53	67.34	0.9163
9.	rs7094179	*CDNF*	65.81	66.46	68.32	0.6485
10.	rs7900873	*CDNF*	67.03	66.49	64.68	0.3912
11.	rs1051730	*CHRNA3*	67.53	65.51	66.58	0.1190
12.	rs16969968	*CHRNA5*	67.45	65.77	66.61	0.2138
13.	rs4680	*COMT*	67.07	66.58	67.62	0.8569
14.	rs135745	*CSNK1E*	65.99	66.63	66.24	0.7121
15.	rs1997644	*CSNK1E*	66.83	66.31	65.68	0.7781
16.	rs1611115	*DBH*	65.74	67.36	70.89	0.0941
17.	rs6271	*DBH*	66.59	66.68	61.00	0.8731
18.	rs4532	*DRD1*	66.55	65.94	67.91	0.9000
19.	rs6277	*DRD2*	66.81	67.06	66.39	0.9148
20.	rs1800497	*DRD2*	66.72	67.70	61.29	0.7106
21.	rs1079597	*DRD2*	67.20	66.38	57.95	0.4397
22.	rs1800498	*DRD2*	67.07	66.46	67.34	0.7979
23.	rs2134655	*DRD3*	65.71	67.81	66.12	0.1250
24.	rs3732790	*DRD3*	67.03	66.42	65.90	0.5267
25.	rs6280	*DRD3*	66.98	66.60	64.46	0.4667
26.	rs963468	*DRD3*	67.25	67.01	65.06	0.5779
27.	rs11246226	*DRD4*	67.31	66.33	66.41	0.4831
28.	rs3758653	*DRD4*	66.51	66.48	69.93	0.9091
***29.***	***rs916455***	***DRD4***	***66.93***	***62.82***	***46.67***	***0.0275***
30.	rs936460	*DRD4*	66.60	66.39	67.70	0.9890
31.	rs3733829	*EGLN2*	66.96	66.76	65.38	0.6238
32.	rs222843	*GABARAP*	66.50	66.09	68.77	0.9562
33.	rs11111	*GDNF*	66.56	65.90	73.85	0.9972
34.	rs1549250	*GDNF*	66.75	65.38	70.34	0.8604
35.	rs1981844	*GDNF*	66.48	66.85	72.22	0.4727
36.	rs2910702	*GDNF*	66.27	66.40	68.79	0.5293
37.	rs2973041	*GDNF*	66.68	66.24	71.95	0.9268
38.	rs2973050	*GDNF*	66.30	66.49	68.89	0.5259
39.	rs3096140	*GDNF*	65.79	66.97	68.52	0.1457
40.	rs3812047	*GDNF*	66.45	67.46	70.87	0.3422
41.	rs6925	*HTR1A*	66.55	67.63	65.63	0.9441
42.	rs1228814	*HTR1B*	67.20	66.47	63.95	0.5336
43.	rs130058	*HTR1B*	67.22	65.55	70.74	0.3419
44.	rs13212041	*HTR1B*	67.09	66.21	66.87	0.5259
45.	rs11568817	*HTR1B*	68.70	65.76	67.12	0.0605
46.	rs6296	*HTR1B*	66.21	67.33	68.60	0.2601
47.	rs6311	*HTR2A*	66.78	66.32	67.41	0.9130
48.	rs6313	*HTR2A*	66.44	66.09	67.44	0.9969
49.	rs6314	*HTR2A*	67.02	63.98	73.67	0.0765
***50.***	***rs7322347***	***HTR2A***	***69.21***	***64.92***	***66.35***	0.0007*
51.	rs7984966	*HTR2A*	67.45	65.65	65.58	0.1356
52.	rs3813929	*HTR2C*	67.01	63.67	66.43	0.2163
53.	rs518147	*HTR2C*	66.73	66.44	67.18	0.3203
54.	rs6318	*HTR2C*	66.43	66.46	67.63	0.2277
55.	rs907094	*PPP1R1B*	67.04	65.18	68.84	0.2994

Nominally significant associations are indicated by bold, italics.

* Significant after Bonferroni correction

^#^ Dominant model (MM vs. Mm and mm).

**Fig 1:**
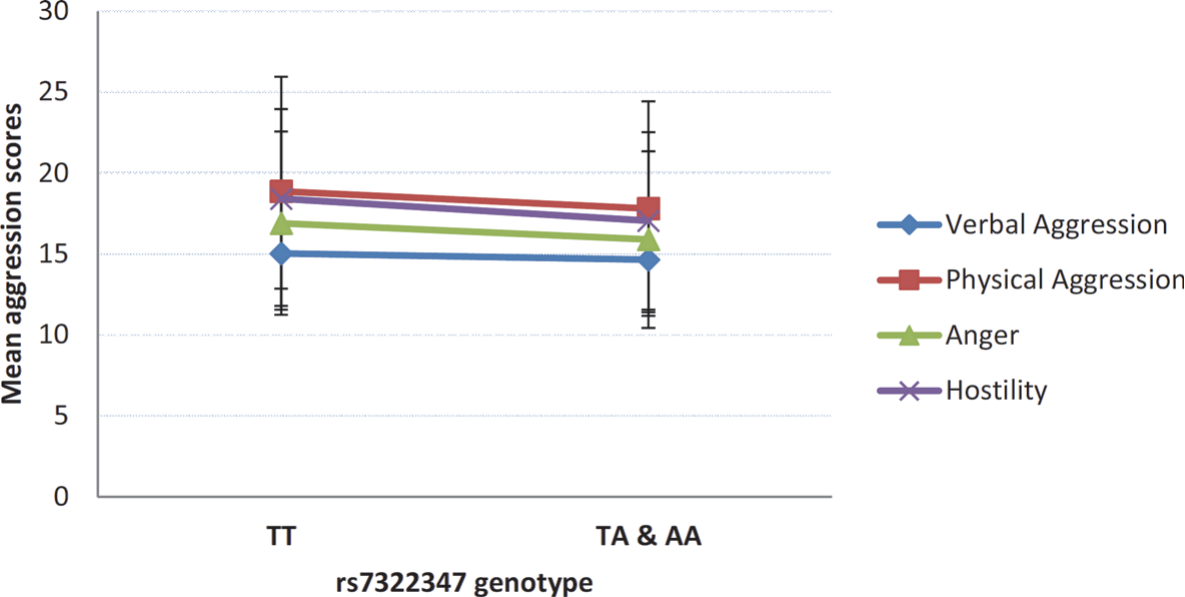
Relationship of each of the Buss-Perry Aggression Questionnaire subscales with rs7322347 A allele carrier status. Mean scores of each the Buss-Perry Aggression Questionnaire subscales according to rs7322347 genotypes. Error bars represent standard errors of the mean.

In order to gain a more detailed insight into the nature of the observed association, *post hoc* analyses were performed testing for possible relationship between rs7322347 and each of the four individual BPAQ subscales (Fig. 1). With the exception of Verbal Aggression, where mean scores did not differ in non-carriers compared to carriers of allele A (15.09±3.47 vs. 14.65±3.24; p = 0.1076), scores of all subscales showed significant association with rs7322347. Differences in mean scores between those homozygous for rs7322347 T and those with at least one copy of rs7322347 A was most remarkable in the case of Hostility (18.41±5.55 vs. 17.05±5.48), with statistical difference between groups virtually equaling that observed with the overall BPAQ scale [F = 11.535, p = 0.0007, η^2^ = 0.015, power = 0.924]. Mean scores for both Physical Aggression and Anger were also higher in the absence of rs7322347 A than in its presence (18.86±7.08 vs. 17.80±6.63) [F = 7.419, p = 0.0066, η^2^ = 0.010, power = 0.776] and (16.91±5.67 vs. 15.89±5.46) [F = 5.858, p = 0.0157, η^2^ = 0.008, power = 0.676], respectively.

### Effect of the HTR2A rs7322347 polymorphism on male and female aggression

**Fig 2:**
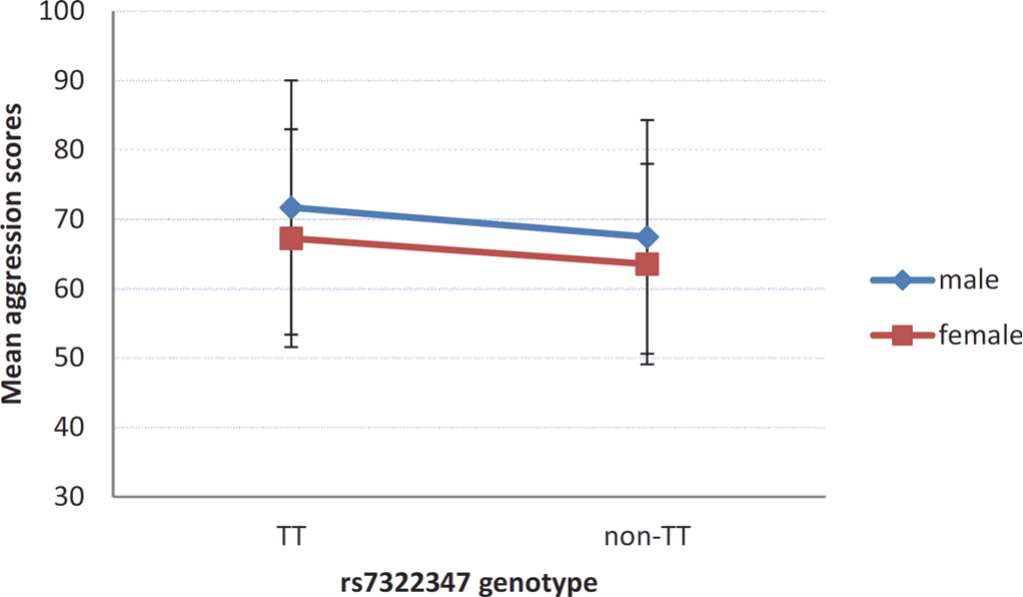
Effect of the HTR2A rs7322347 polymorphism on male and female aggression. Mean scores of the Buss-Perry Aggression Questionnaire in males and females according to rs7322347 genotypes. Error bars represent standard errors of the mean.

As significant gender effect was observed in the BPAQ scores, male vs. female differences were also tested in terms of rs7322347 genotype and aggression using two-way ANCOVA with age as covariate. Although interaction between gender and aggression scores was highly significant [F = 10.991, p = 0.0010, η^2^ = 0.014, power = 0.912], no gene-sex interaction was found (p = 0.8834). Both males and females carrying the minor (A) allele of rs7322347 showed lower levels of aggression (Fig. 2).

### Linkage disequilibrium (LD) and haplotype analyses within the HTR2A gene

**Fig 3:**
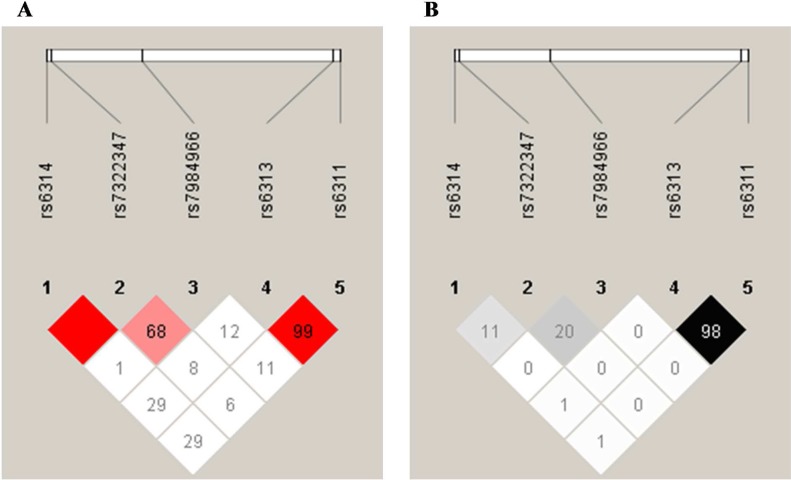
Linkage disequilibrium patterns between SNPs studies within the HTR2A gene. A: Lewontin’s D’ (%) and B: r2 (%) values of linkage disequilibrium between each SNP pairs, as determined by HaploView (version 4.2.). Higher values and darker colors indicate stronger LD between loci pairs. Red square indicates 100% LD.

Taken that four other SNPs than rs7322347 (rs6311 C/T, rs6313 G/A, rs6314 G/A and rs7984966 T/C) within the *HTR2A* gene were also genotyped in this study, LD and haplotype analyses were performed as well to explore possible further contribution of loci in nearby regions to higher aggression levels. The associating polymorphism rs7322347 was found to be in complete linkage disequilibrium (D’ = 1) with rs6314 located 1069 bp upstream from rs7322347 (Fig. 3), due to the fact that allele A of rs6314 could only be observed in subjects also carrying rs7322347 A and that all individuals homozygous for rs6314 A were homozygous for rs7322347 A as well. However, this was accompanied by a relatively low r^2^ value as there was a marked difference in MAFs for these two SNPs (8.8% for rs6314 vs. 44.2% for rs7322347). The polymorphism rs7322347 was in strong LD with rs7984966 as well (chromosomal distance: 19343 bp), although to a lesser extent than with rs6314. In addition, prominently high LD was also observed between rs6313 and rs6311 spaced 1538 bp apart, where in the majority of cases allele A of rs6313 was linked to rs6311 T (662/665 chromosomes; 99.6%) and allele G of rs6313 to rs6311 C (850/853 chromosomes; 99.7%) (Fig. 3).

One-way ANCOVAs were applied on the overall BPAQ scale scores with 2-SNP haplotypes (comprising rs7322347 and each of the other four *HTR2A* variants genotyped) as the grouping variable and gender and age as covariates (Table 3). In a dominant model (haplotypes containing only major alleles of the constituting SNPs), haplotypes rs6314/ rs7322347 and rs7322347/ rs7984966 showed a significant effect [F = 11.128, p = 0.0009, η^2^ = 0.014, power = 0.915 and F = 7.352, p = 0.0068, η^2^ = 0.009, power = 0.773, respectively], while no significant differences in the mean scores of aggression were observed with regard to the other two haplotypes analyzed (p = 0.1875 and p = 0.1232, respectively). Subjects homozygous for haplotype rs6314 G/ rs7322347 T had higher aggression scores as compared to the rest of the population (69.05±17.07 vs. 65.33±15.77). Similarly, individuals carrying haplotype rs7322347 T/ rs7984966 T on both chromosomes presented with higher mean BPAQ scores than those with other haplotype combinations (68.83±17.19 vs. 65.64±15.85). Haplotype-wise analyses also indicated significant association of haplotype rs6314/ rs7322347, but to a lesser extent than in the dominant model [F = 3.205, p = 0.0408, η^2^ = 0.004, power = 0.614] (Table 4).

**Table 3:** Association of rs7322347 comprising 2-SNP within-*HTR2A* haplotypes with aggression scores.

	Aggression score		p
	HH	Hh & hh	
**rs6314/ rs7322347**	69.05±17.07	65.33±15.77	***0.0009***
**rs7322347/ rs7984966**	68.83±17.19	65.64±15.85	***0.0068***
**rs7322347/ rs6313**	68.58±18.92	66.23±15.89	0.1875
**rs7322347/ rs6311**	68.95±18.69	66.19±15.92	0.1232

H: Haplotype containing major alleles of the constituting SNPs;

h: haplotype containing minor allele of at least of the two constituting SNPs

Significant associations are indicated by bold, italics.

**Table 4:** Haplotype-wise analysis of rs7322347 and each of the other *HTR2A* SNPs studied.

	N	Haplotype frequency	Aggression score	p
rs6314G-rs7322347T	862	0,56	67.28±16.53	***0.041***
rs6314G-rs7322347A	547	0,35	65.78±16.04	
rs6314A-rs7322347A	135	0,09	64.46±15.28	
rs6314A-rs7322347T	0	0	-	
rs7322347T-rs7984966T	809	0,52	67.34±16.54	0.115
rs7322347A-rs7984966C	347	0,22	65.45±15.63	
rs7322347A-rs7984966T	335	0,22	65.59±16.18	
rs7322347T-rs7984966C	53	0,03	66.37±16.38	
rs7322347T-rs6313G	592	0,38	67.03±16.50	0.072
rs7322347A-rs6313A	405	0,26	65.88±16.40	
rs7322347A-rs6313G	277	0,18	64.99±15.14	
rs7322347T-rs6313A	270	0,17	67.83±16.60	
rs7322347T-rs6311C	590	0,38	67.07±16.48	0.074
rs7322347A-rs6311T	403	0,26	65.92±16.42	
rs7322347A-rs6311C	279	0,18	64.94±15.10	
rs7322347T-rs6311T	272	0,18	67.74±16.66	

Significant p value is indicated by bold, italics.

## Discussion

In this study, we examined possible contribution of 55 SNPs to aggressive tendencies measured by the BPAQ in the general adult Hungarian population [33,34]. Only two of these SNPs showed association reaching nominal significance, and merely rs7322347 of the *HTR2A* gene retained significant effect after Bonferroni adjustment.

These findings underpin the long-suspected key role of the serotonin neurotransmitter system in aggression and the related disorders [40,41]. There is convergent evidence that low or impaired serotonergic function underlies aggression and impulsivity [42–44]. As within the central nervous system (CNS) serotonin is synthesized solely in neurons of the raphe nuclei innervating virtually the entire neuraxis, this neurotransmitter is believed to exert a global effect on the brain with a holistically general role, even though local specialized functions are achieved by a variety of receptors [45,46]. It has been proposed that the principal role of serotonin might be the withdrawal from dangerous and aversive situations; consequently, serotonergic hypofunction could lead to impaired avoidance of undesirable stimuli, which in turn could provoke aggressive responses [47]. Strong experimental evidence supports this concept. The inverse correlation of aggression, impulsivity and antisocial behavior with serotonin metabolite 5-hydroxyindoleacetic acid levels in the cerebrospinal fluid was already known decades ago [40,48–50]. Later on, numerous studies confirmed these early observations regarding the relationship between dysregulation of the serotonergic system and aggressive-impulsive traits both in human and animals [51–54]. Behavioral functions of serotonin and also the effect of drugs influencing serotonergic mechanisms shows a marked conservation even between evolutionarily remote species [55]. This enables utilization of animal models for different types of aggression, e.g. affective (or defensive) and predatory (referred to as impulsive and premediated in humans, respectively) [56]. Data especially on rodents and felines provide valuable insight into underlying molecular mechanisms, shedding light for example on the interplay of proinflammatory cytokines and serotonin receptors in defensive rage and also on differential modulation of aggression by distinct types of serotonin receptors [57–59]. Administration of selective serotonin reuptake inhibitors (SSRIs), such as fluoxetine, citalopram or paroxetine usually reduces aggression [60–69], though contradictory results have also been reported, especially in juvenile humans and animals [70–72]. Reduced levels of serotonin caused by depletion of its precursor tryptophan have been linked to aggressive behavior [73–76], and disrupted function of enzymes involved in serotonin metabolism, such as tryptophan hydroxylase or monoamine oxidase, are also related to aggressive traits [77–79]. Observations on the link between life history of aggression and platelet serotonin content as well as platelet serotonin receptor and transporter binding further underpin the constantly growing body of evidence referring to a close relationship between the serotonergic system and aggression [80–82].

Serotonin (5-hydroxytryptamine) receptor 2a, encoded by the gene *HTR2A*, is a G-protein coupled excitatory receptor exerting its influence through the activation of secondary messengers phospholipase C and D [83]. Among others it is expressed in high levels on pyramidal cells of the prefrontal cortex, where it is ideally positioned to modulate both cognitive functions such as working memory or executive control and also emotions through dynamic interactions with the amygdala [84,85]. Serotonin receptors are also distributed along the midbrain periaqueductal grey (PAG) and the hypothalamus [56], brain areas that both have a direct connection with the prefrontal cortex and amygdala and long have been proved to control components of aggression including vocalization [86,87]. In accordance, mice with inherited aberrations in development and function of serotonergic neurons in the CNS exhibit increased levels of aggression which can be ameliorated by SSRIs [88]. Functional polymorphisms of the *HTR2A* gene are thus expected to influence neuronal networks regulating all the above mentioned features, providing a physiological basis for associations between *HTR2A* genetic variations and different mental states. During the last decade, several groups investigated SNPs of the *HTR2A* gene in connection with psychiatric and personality disorders [89–95]. Noteworthy observations have been made with regard to a number of variations located mainly in the promoter or the coding region; however, though scarce, literature data also indicate that intronic variant rs7322347 might as well be of interest from behavioral aspects, as it showed marked association with the combined subtype of childhood attention-deficit hyperactivity disorder (ADHD) and with suicide attempt in females subjected to physical assault in younger age [96,97].

Interestingly, according to our findings the missense polymorphism rs6314 is in complete LD with rs7322347, and the haplotype defined by these two SNPs has a similarly high impact on aggression levels as rs7322347 alone, despite the great difference observed between their MAFs. This might reflect that a complex background lies behind the robust association observed in the case of rs7322347, possibly consisting of several minor factors. Intrinsically, marked physiological effect of a single genetic variation with a MAF nearing 50% is generally improbable, simply based on the consideration that the spread of newly arisen alleles with functional relevance is most probably controlled by either positive or negative selection, hardly allowing quasi equal allele frequencies to evolve. Although in the present case it is plausible that a fine evolutionary balance has been struck between avoiding fights thus injury and gaining access to better resources, it cannot be excluded that other, linked polymorphic loci also contribute to the overall observed effect, even though similarly high D’ values as seen for rs6314 are unlikely for any such sites. Indeed, full linkage disequilibrium can only be expected when no crossing over event between the linked loci has yet occurred, which is mainly characteristic to the situation when at least one of the polymorphic sites is evolutionarily young. It is, though, noteworthy that immensely strong LD has been identified elsewhere within the *HTR2A* gene as well (between rs6311 and rs6313), both in this study and before [98–100].

As the linked polymorphism rs6314 causes a histidine to tyrosine change, thus the substitution of a basic amino acid residue to an uncharged one, this SNP could potentially affect both protein structure and function [101]. *In vitro* studies implicate that its rare allele causes slower receptor response, decreased activation levels of phospholipases C and D, reduced calcium ion mobilization and thus a general hypofunctioning of the whole signaling cascade [102,103]. Recent findings imply that rs6314 also interferes with adequate splicing of pre-mRNA, with defective transcript forms triggering the RNA surveillance machinery, leading to a lower expression of the variant allele both on RNA and protein level [104].

Another possible explanation for the observed relationship between rs7322347 and aggression lies in gene regulation. Over the last few years, growing number of disease-associating polymorphisms in intergenic and intronic regions identified especially in GWA studies, combined with the fact that the more complex an organism is, the larger proportion of its genome will consist of non-coding sequences, has drawn attention of the scientific community towards the significance of expression regulation. By now, light has been thrown on several molecular mechanisms modifying gene expression, mostly with the involvement of non-coding sequences. Polymorphic intronic sites can lead to splicing efficiency bias or modified pre-mRNA stability, or they might affect long-distance gene regulation, for instance as part of an enhancer or an insulator, or through the RNAi pathway. In fact, according to the miRBase registry, T allele of rs7322347 disrupts a potential miRNA binding site [105,106]. It has recently been demonstrated by our group that differences in transcriptional regulation caused by a miRNA binding site disrupting SNP can indeed contribute to elevated aggression levels [107]. Though functional relevance of intronic miRNA target sites is obscure, recent evidence suggests that at least in plants miRNA interaction with intronic sequences is indeed involved in gene regulation processes [108]. In addition, expression quantitative trait loci (eQTL) data (http://genenetwork.nl/bloodeqtlbrowser) indicate that minor allele (A) of rs7322347 negatively affects (Z-score: -8.06) transcription of the *ESD* gene located 34 kb downstream of *HTR2A* [109]. *ESD* encodes esterase D, a poorly characterized protein with a suggested role in the recycling of sialic acids and also in detoxification [110,111]. Thus, it would be intriguing to explore possible interaction of *ESD* with neurobiological aspects and behavioral traits, especially as it is expressed all across the brain in considerable amounts according to AceView and TiGER databases [112,113].

In the present report, we demonstrate a robust contribution of the rs7322347 variation within the gene encoding serotonin receptor 2a to aggressive traits. As the study was conducted on a large sample of 887 normal individuals and the effect of this polymorphism was strong enough to endure Bonferroni correction for multiple tests, it can be assumed that the observed association has a substantive biological basis. This might provide us with a better insight into the driving forces underlying aggression, hopefully facilitating early identification of individuals at risk, hereby also improving prevention of negative consequences derived from aggressive manifestations. Nevertheless, care must be taken not to overestimate the impact of these findings. Psychological and behavioral processes are complex traits comprising a not at all negligible environmental component, and deciphering all gene-environment (G×E) as well as epistatic interactions is more than challenging. There is far more to mental states than simply biochemical processes; thus, even though anatomical structure of the brain, neurophysiological functioning and gene expression regulation mechanisms are essentially identical in all human beings, socialization and culture also largely influences our acts and behavior [114,115]. Presumably, the role of genes in terms of human behavior is neither less nor more than establishing a reaction spectrum; within the available range, however, former experiences, belief-systems and social atmosphere are supposed to serve as key determinants of the actual behavior [46].

In conclusion, this study adds on the growing evidence that the serotoninergic system greatly influences aggressive tendencies. To our best knowledge, this is the first report demonstrating a direct relationship between the *HTR2A* gene and aggression. However, confirmation of the present findings by independent replication would inevitably be necessary before drawing any further conclusions from these results. Functional studies should also be performed in order to explore the exact biochemical background of the association described, and to elicit possible contribution of rs7322347 to psychiatric and personality disorders. By no means forgetting about the significance of environmental exposure, our findings will hopefully provide help to elucidate the genetic basis behind increased predisposition to aggression.

## References

[1] Krug EG, Mercy JA, Dahlberg LL, Zwi AB (2002) The world report on violence and health. Lancet 360: 1083–1088. 12384003 10.1016/S0140-6736(02)11133-0

[2] Swann AC (2003) Neuroreceptor mechanisms of aggression and its treatment. J Clin Psychiatry 64 Suppl 4: 26–35. 12672262

[3] Zahodne LB, Ornstein K, Cosentino S, Devanand DP, Stern Y (2013) Longitudinal Relationships Between Alzheimer Disease Progression and Psychosis, Depressed Mood, and Agitation/Aggression. Am J Geriatr Psychiatry S1064–7481(13)00201–7 [pii] 10.1016/j.jagp.2013.03.014. 10.1016/j.jagp.2013.03.014 PMC3858495 23871118

[4] Kachadourian LK, Homish GG, Quigley BM, Leonard KE (2012) Alcohol expectancies, alcohol use, and hostility as longitudinal predictors of alcohol-related aggression. Psychol Addict Behav 26: 414–422. 10.1037/a0025842 22004128 PMC4030542

[5] Latalova K (2014) Violence and duration of untreated psychosis in first-episode patients. Int J Clin Pract 68: 330–335. 10.1111/ijcp.12327 24471741

[6] Volavka J (2013) Violence in schizophrenia and bipolar disorder. Psychiatr Danub 25: 24–33. 23470603

[7] Wexler E (2013) Clinical neurogenetics: behavioral management of inherited neurodegenerative disease. Neurol Clin 31: 1121–1144. 10.1016/j.ncl.2013.04.016 24176427

[8] Georgiev AV, Klimczuk AC, Traficonte DM, Maestripieri D (2013) When violence pays: a cost-benefit analysis of aggressive behavior in animals and humans. Evol Psychol 11: 678–699. 23864299 10.1177/147470491301100313 PMC3859192

[9] Dijkstra JK, Lindenberg S, Zijlstra L, Bouma E, Veenstra R (2013) The secret ingredient for social success of young males: a functional polymorphism in the 5HT2A serotonin receptor gene. PLoS One 8: e54821 10.1371/journal.pone.0054821 23457454 PMC3573014

[10] Maynard Smith J, Harper DG (1988) The evolution of aggression: can selection generate variability? Philos Trans R Soc Lond B Biol Sci 319: 557–570. 2905492 10.1098/rstb.1988.0065

[11] Cairns RB (1996) Aggression from a developmental perspective: genes, environments and interactions. Ciba Found Symp 194: 45–56; discussion 57–60. 8862869 10.1002/9780470514825.ch3

[12] Niv S, Tuvblad C, Raine A, Baker LA (2013) Aggression and Rule-breaking: Heritability and stability of antisocial behavior problems in childhood and adolescence. J Crim Justice 41 10.1016/j.jcrimjus.2013.06.014 24347737 PMC3856338

[13] Brendgen M, Vitaro F, Boivin M, Dionne G, Perusse D (2006) Examining genetic and environmental effects on reactive versus proactive aggression. Dev Psychol 42: 1299–1312. 17087562 10.1037/0012-1649.42.6.1299

[14] Baker LA, Raine A, Liu J, Jacobson KC (2008) Differential genetic and environmental influences on reactive and proactive aggression in children. J Abnorm Child Psychol 36: 1265–1278. 10.1007/s10802-008-9249-1 18615267 PMC2609906

[15] Tuvblad C, Raine A, Zheng M, Baker LA (2009) Genetic and environmental stability differs in reactive and proactive aggression. Aggress Behav 35: 437–452. 10.1002/ab.20319 19688841 PMC2771207

[16] Bezdjian S, Tuvblad C, Raine A, Baker LA (2011) The genetic and environmental covariation among psychopathic personality traits, and reactive and proactive aggression in childhood. Child Dev 82: 1267–1281. 10.1111/j.1467-8624.2011.01598.x 21557742 PMC3134591

[17] Rhee SH, Waldman ID (2002) Genetic and environmental influences on antisocial behavior: a meta-analysis of twin and adoption studies. Psychol Bull 128: 490–529. 12002699

[18] Lesch KP (2005) Serotonergic gene inactivation in mice: models for anxiety and aggression? Novartis Found Symp 268: 111–140; discussion 140–116, 167–170. 16206878

[19] Davidson RJ, Putnam KM, Larson CL (2000) Dysfunction in the neural circuitry of emotion regulation—a possible prelude to violence. Science 289: 591–594. 10915615 10.1126/science.289.5479.591

[20] Pavlov KA, Chistiakov DA, Chekhonin VP (2012) Genetic determinants of aggression and impulsivity in humans. J Appl Genet 53: 61–82. 10.1007/s13353-011-0069-6 21994088

[21] Craig IW, Halton KE (2009) Genetics of human aggressive behaviour. Hum Genet 126: 101–113. 10.1007/s00439-009-0695-9 19506905

[22] Albaugh MD, Harder VS, Althoff RR, Rettew DC, Ehli EA, et al (2010) COMT Val158Met genotype as a risk factor for problem behaviors in youth. J Am Acad Child Adolesc Psychiatry 49: 841–849. 10.1016/j.jaac.2010.05.015 20643317 PMC3141335

[23] Kuepper Y, Grant P, Wielpuetz C, Hennig J (2013) MAOA-uVNTR genotype predicts interindividual differences in experimental aggressiveness as a function of the degree of provocation. Behav Brain Res 247: 73–78. 10.1016/j.bbr.2013.03.002 23499704

[24] Grigorenko EL, De Young CG, Eastman M, Getchell M, Haeffel GJ, et al (2010) Aggressive behavior, related conduct problems, and variation in genes affecting dopamine turnover. Aggress Behav 36: 158–176. 10.1002/ab.20339 20127808

[25] Turecki G, Briere R, Dewar K, Antonetti T, Lesage AD, et al (1999) Prediction of level of serotonin 2A receptor binding by serotonin receptor 2A genetic variation in postmortem brain samples from subjects who did or did not commit suicide. Am J Psychiatry 156: 1456–1458. 10484964 10.1176/ajp.156.9.1456

[26] Aslund C, Comasco E, Nordquist N, Leppert J, Oreland L, et al (2013) Self-reported family socioeconomic status, the 5-HTTLPR genotype, and delinquent behavior in a community-based adolescent population. Aggress Behav 39: 52–63. 10.1002/ab.21451 22987641

[27] Terranova C, Tucci M, Sartore D, Cavarzeran F, Barzon L, et al (2012) Alcohol dependence and criminal behavior: preliminary results of an association study of environmental and genetic factors in an Italian male population. J Forensic Sci 57: 1343–1348. 10.1111/j.1556-4029.2012.02243.x 22881191

[28] Aluja A, Garcia LF, Blanch A, Fibla J (2011) Association of androgen receptor gene, CAG and GGN repeat length polymorphism and impulsive-disinhibited personality traits in inmates: the role of short-long haplotype. Psychiatr Genet 21: 229–239. 10.1097/YPG.0b013e328345465e 21368712

[29] Kotyuk E, Keszler G, Nemeth N, Ronai Z, Sasvari-Szekely M, et al (2013) Glial cell line-derived neurotrophic factor (GDNF) as a novel candidate gene of anxiety. PLoS One 8: e80613 10.1371/journal.pone.0080613 24324616 PMC3855631

[30] Siddiq A, Aminova LR, Troy CM, Suh K, Messer Z, et al (2009) Selective inhibition of hypoxia-inducible factor (HIF) prolyl-hydroxylase 1 mediates neuroprotection against normoxic oxidative death via HIF- and CREB-independent pathways. J Neurosci 29: 8828–8838. 10.1523/JNEUROSCI.1779-09.2009 19587290 PMC3290095

[31] Bryant CD, Parker CC, Zhou L, Olker C, Chandrasekaran RY, et al (2012) Csnk1e is a genetic regulator of sensitivity to psychostimulants and opioids. Neuropsychopharmacology 37: 1026–1035. 10.1038/npp.2011.287 22089318 PMC3280656

[32] Xu H, Belkacemi L, Jog M, Parrent A, Hebb MO (2013) Neurotrophic factor expression in expandable cell populations from brain samples in living patients with Parkinson's disease. FASEB J 27: 4157–4168. 10.1096/fj.12-226555 23825231

[33] Buss AH, Perry M (1992) The aggression questionnaire. J Pers Soc Psychol 63: 452–459. 1403624 10.1037//0022-3514.63.3.452

[34] Gerevich J, Bacskai E, Czobor P (2007) The generalizability of the Buss-Perry Aggression Questionnaire. Int J Methods Psychiatr Res 16: 124–136. 17849418 10.1002/mpr.221 PMC6878225

[35] Boor K, Ronai Z, Nemoda Z, Gaszner P, Sasvari-Szekely M, et al (2002) Noninvasive genotyping of dopamine receptor D4 (DRD4) using nanograms of DNA from substance-dependent patients. Curr Med Chem 9: 793–797. 11966444 10.2174/0929867024606821

[36] Sherry ST, Ward MH, Kholodov M, Baker J, Phan L, et al (2001) dbSNP: the NCBI database of genetic variation. Nucleic Acids Res 29: 308–311. 11125122 10.1093/nar/29.1.308 PMC29783

[37] Barrett JC, Fry B, Maller J, Daly MJ (2005) Haploview: analysis and visualization of LD and haplotype maps. Bioinformatics 21: 263–265. 15297300 10.1093/bioinformatics/bth457

[38] Stephens M, Donnelly P (2003) A comparison of bayesian methods for haplotype reconstruction from population genotype data. Am J Hum Genet 73: 1162–1169. 14574645 10.1086/379378 PMC1180495

[39] Stephens M, Smith NJ, Donnelly P (2001) A new statistical method for haplotype reconstruction from population data. Am J Hum Genet 68: 978–989. 11254454 10.1086/319501 PMC1275651

[40] Brown GL, Goodwin FK, Ballenger JC, Goyer PF, Major LF (1979) Aggression in humans correlates with cerebrospinal fluid amine metabolites. Psychiatry Res 1: 131–139. 95232 10.1016/0165-1781(79)90053-2

[41] Brown GL, Linnoila MI (1990) CSF serotonin metabolite (5-HIAA) studies in depression, impulsivity, and violence. J Clin Psychiatry 51 Suppl: 31–41; discussion 42–33. 1691169

[42] Stanley B, Molcho A, Stanley M, Winchel R, Gameroff MJ, et al (2000) Association of aggressive behavior with altered serotonergic function in patients who are not suicidal. Am J Psychiatry 157: 609–614. 10739421 10.1176/appi.ajp.157.4.609

[43] Winstanley CA (2011) Gambling rats: insight into impulsive and addictive behavior. Neuropsychopharmacology 36: 359 10.1038/npp.2010.167 21116252 PMC3055520

[44] Pattij T, Vanderschuren LJ (2008) The neuropharmacology of impulsive behaviour. Trends Pharmacol Sci 29: 192–199. 10.1016/j.tips.2008.01.002 18304658

[45] Azmitia EC (2007) Serotonin and brain: evolution, neuroplasticity, and homeostasis. Int Rev Neurobiol 77: 31–56. 17178471 10.1016/S0074-7742(06)77002-7

[46] Yildirim BO, Derksen JJ (2013) Systematic review, structural analysis, and new theoretical perspectives on the role of serotonin and associated genes in the etiology of psychopathy and sociopathy. Neurosci Biobehav Rev 37: 1254–1296. 10.1016/j.neubiorev.2013.04.009 23644029

[47] Tops M, Russo S, Boksem MA, Tucker DM (2009) Serotonin: modulator of a drive to withdraw. Brain Cogn 71: 427–436. 10.1016/j.bandc.2009.03.009 19423206

[48] Kruesi MJ, Rapoport JL, Hamburger S, Hibbs E, Potter WZ, et al (1990) Cerebrospinal fluid monoamine metabolites, aggression, and impulsivity in disruptive behavior disorders of children and adolescents. Arch Gen Psychiatry 47: 419–426. 1691910 10.1001/archpsyc.1990.01810170019003

[49] Linnoila M, Virkkunen M, Scheinin M, Nuutila A, Rimon R, et al (1983) Low cerebrospinal fluid 5-hydroxyindoleacetic acid concentration differentiates impulsive from nonimpulsive violent behavior. Life Sci 33: 2609–2614. 6198573 10.1016/0024-3205(83)90344-2

[50] Coccaro EF, Lee R (2010) Cerebrospinal fluid 5-hydroxyindolacetic acid and homovanillic acid: reciprocal relationships with impulsive aggression in human subjects. Journal of neural transmission 117: 241–248. 10.1007/s00702-009-0359-x 20069438

[51] Manuck SB, Flory JD, McCaffery JM, Matthews KA, Mann JJ, et al (1998) Aggression, impulsivity, and central nervous system serotonergic responsivity in a nonpatient sample. Neuropsychopharmacology 19: 287–299. 9718592 10.1016/S0893-133X(98)00015-3

[52] Fairbanks LA, Melega WP, Jorgensen MJ, Kaplan JR, McGuire MT (2001) Social impulsivity inversely associated with CSF 5-HIAA and fluoxetine exposure in vervet monkeys. Neuropsychopharmacology 24: 370–378. 11182532 10.1016/S0893-133X(00)00211-6

[53] Mosienko V, Bert B, Beis D, Matthes S, Fink H, et al (2012) Exaggerated aggression and decreased anxiety in mice deficient in brain serotonin. Transl Psychiatry 2: e122 10.1038/tp.2012.44 22832966 PMC3365263

[54] Cases O, Seif I, Grimsby J, Gaspar P, Chen K, et al (1995) Aggressive behavior and altered amounts of brain serotonin and norepinephrine in mice lacking MAOA. Science 268: 1763–1766. 7792602 10.1126/science.7792602 PMC2844866

[55] Herculano AM, Maximino C (2014) Serotonergic modulation of zebrafish behavior: Towards a paradox. Prog Neuropsychopharmacol Biol Psychiatry S0278–5846(14)00061-X [pii] 10.1016/j.pnpbp.2014.03.008. 10.1016/j.pnpbp.2014.03.008 24681196

[56] Siegel A, Bhatt S, Bhatt R, Zalcman SS (2007) The neurobiological bases for development of pharmacological treatments of aggressive disorders. Current neuropharmacology 5: 135–147. 18615178 10.2174/157015907780866929 PMC2435345

[57] Hassanain M, Bhatt S, Zalcman S, Siegel A (2005) Potentiating role of interleukin-1beta (IL-1beta) and IL-1beta type 1 receptors in the medial hypothalamus in defensive rage behavior in the cat. Brain research 1048: 1–11. 15919060 10.1016/j.brainres.2005.04.086

[58] Hassanain M, Bhatt S, Siegel A (2003) Differential modulation of feline defensive rage behavior in the medial hypothalamus by 5-HT1A and 5-HT2 receptors. Brain research 981: 201–209. 12885442 10.1016/s0006-8993(03)03036-1

[59] Siegel A, Roeling TA, Gregg TR, Kruk MR (1999) Neuropharmacology of brain-stimulation-evoked aggression. Neurosci Biobehav Rev 23: 359–389. 9989425 10.1016/s0149-7634(98)00040-2

[60] Vartiainen H, Tiihonen J, Putkonen A, Koponen H, Virkkunen M, et al (1995) Citalopram, a selective serotonin reuptake inhibitor, in the treatment of aggression in schizophrenia. Acta Psychiatr Scand 91: 348–351. 7639092 10.1111/j.1600-0447.1995.tb09793.x

[61] Ho HP, Olsson M, Westberg L, Melke J, Eriksson E (2001) The serotonin reuptake inhibitor fluoxetine reduces sex steroid-related aggression in female rats: an animal model of premenstrual irritability? Neuropsychopharmacology 24: 502–510. 11282250 10.1016/S0893-133X(00)00219-0

[62] Ten Eyck GR, Regen EM (2014) Chronic fluoxetine treatment promotes submissive behavior in the territorial frog, Eleutherodactylus coqui. Pharmacol Biochem Behav 124: 86–91. 10.1016/j.pbb.2014.05.018 24887449

[63] Fanning JR, Berman ME, Guillot CR, Marsic A, McCloskey MS (2014) Serotonin (5-HT) augmentation reduces provoked aggression associated with primary psychopathy traits. J Pers Disord 28: 449–461. 10.1521/pedi_2012_26_065 22984854

[64] Berman ME, McCloskey MS, Fanning JR, Schumacher JA, Coccaro EF (2009) Serotonin augmentation reduces response to attack in aggressive individuals. Psychol Sci 20: 714–720. 10.1111/j.1467-9280.2009.02355.x 19422623 PMC2728471

[65] Coccaro EF, Kavoussi RJ, Hauger RL (1997) Serotonin function and antiaggressive response to fluoxetine: a pilot study. Biol Psychiatry 42: 546–552. 9376450 10.1016/S0006-3223(97)00309-0

[66] Coccaro EF, Kavoussi RJ (1997) Fluoxetine and impulsive aggressive behavior in personality-disordered subjects. Arch Gen Psychiatry 54: 1081–1088. 9400343 10.1001/archpsyc.1997.01830240035005

[67] Coccaro EF, Lee RJ, Kavoussi RJ (2009) A double-blind, randomized, placebo-controlled trial of fluoxetine in patients with intermittent explosive disorder. J Clin Psychiatry 70: 653–662. 10.4088/JCP.08m04150 19389333

[68] Phan KL, Lee R, Coccaro EF (2011) Personality predictors of antiaggressive response to fluoxetine: inverse association with neuroticism and harm avoidance. International clinical psychopharmacology 26: 278–283. 10.1097/YIC.0b013e32834978ac 21795983

[69] Coccaro EF, Lee R, Kavoussi RJ (2010) Aggression, suicidality, and intermittent explosive disorder: serotonergic correlates in personality disorder and healthy control subjects. Neuropsychopharmacology 35: 435–444. 10.1038/npp.2009.148 19776731 PMC3055394

[70] Kiryanova V, Dyck RH (2014) Increased aggression, improved spatial memory, and reduced anxiety-like behaviour in adult male mice exposed to fluoxetine early in life. Dev Neurosci 36: 396–408. 10.1159/000363102 25115143

[71] Constantino JN, Liberman M, Kincaid M (1997) Effects of serotonin reuptake inhibitors on aggressive behavior in psychiatrically hospitalized adolescents: results of an open trial. J Child Adolesc Psychopharmacol 7: 31–44. 9192540 10.1089/cap.1997.7.31

[72] Ricci LA, Melloni RH Jr., (2012) Repeated fluoxetine administration during adolescence stimulates aggressive behavior and alters serotonin and vasopressin neural development in hamsters. Behav Neurosci 126: 640–653. 10.1037/a0029761 23025830

[73] Passamonti L, Crockett MJ, Apergis-Schoute AM, Clark L, Rowe JB, et al (2012) Effects of acute tryptophan depletion on prefrontal-amygdala connectivity while viewing facial signals of aggression. Biol Psychiatry 71: 36–43. 10.1016/j.biopsych.2011.07.033 21920502 PMC3368260

[74] Robinson OJ, Cools R, Sahakian BJ (2012) Tryptophan depletion disinhibits punishment but not reward prediction: implications for resilience. Psychopharmacology (Berl) 219: 599–605. 10.1007/s00213-011-2410-5 21769566 PMC3249152

[75] Crockett MJ, Clark L, Tabibnia G, Lieberman MD, Robbins TW (2008) Serotonin modulates behavioral reactions to unfairness. Science 320: 1739 10.1126/science.1155577 18535210 PMC2504725

[76] McCloskey MS, Ben-Zeev D, Lee R, Berman ME, Coccaro EF (2009) Acute tryptophan depletion and self-injurious behavior in aggressive patients and healthy volunteers. Psychopharmacology (Berl) 203: 53–61. 10.1007/s00213-008-1374-6 18946662

[77] Angoa-Perez M, Kane MJ, Sykes CE, Perrine SA, Church MW, et al (2014) Brain serotonin determines maternal behavior and offspring survival. Genes Brain Behav 13: 579–591. 10.1111/gbb.12159 25077934 PMC4804711

[78] Godar SC, Bortolato M, Castelli MP, Casti A, Casu A, et al (2014) The aggression and behavioral abnormalities associated with monoamine oxidase A deficiency are rescued by acute inhibition of serotonin reuptake. J Psychiatr Res 56: 1–9. 10.1016/j.jpsychires.2014.04.014 24882701 PMC4114985

[79] Alia-Klein N, Goldstein RZ, Kriplani A, Logan J, Tomasi D, et al (2008) Brain monoamine oxidase A activity predicts trait aggression. J Neurosci 28: 5099–5104. 10.1523/JNEUROSCI.0925-08.2008 18463263 PMC2430409

[80] Marseille R, Lee R, Coccaro EF (2012) Inter-relationship between different platelet measures of 5-HT and their relationship to aggression in human subjects. Prog Neuropsychopharmacol Biol Psychiatry 36: 277–281. 10.1016/j.pnpbp.2011.10.004 22019855

[81] Coccaro EF, Lee R, Kavoussi RJ (2010) Inverse relationship between numbers of 5-HT transporter binding sites and life history of aggression and intermittent explosive disorder. J Psychiatr Res 44: 137–142. 10.1016/j.jpsychires.2009.07.004 19767013

[82] Goveas JS, Csernansky JG, Coccaro EF (2004) Platelet serotonin content correlates inversely with life history of aggression in personality-disordered subjects. Psychiatry Res 126: 23–32. 15081624 10.1016/j.psychres.2004.01.006

[83] Fink KB, Gothert M (2007) 5-HT receptor regulation of neurotransmitter release. Pharmacol Rev 59: 360–417. 18160701 10.1124/pr.107.07103

[84] Weber ET, Andrade R (2010) Htr2a Gene and 5-HT(2A) Receptor Expression in the Cerebral Cortex Studied Using Genetically Modified Mice. Front Neurosci 4 10.3389/fnins.2010.00300 20802802 PMC2928707

[85] Salzman CD, Fusi S (2010) Emotion, cognition, and mental state representation in amygdala and prefrontal cortex. Annu Rev Neurosci 33: 173–202. 10.1146/annurev.neuro.051508.135256 20331363 PMC3108339

[86] Kruk MR, Van der Poel AM, Meelis W, Hermans J, Mostert PG, et al (1983) Discriminant analysis of the localization of aggression-inducing electrode placements in the hypothalamus of male rats. Brain research 260: 61–79. 6681724 10.1016/0006-8993(83)90764-3

[87] Ferris CF, Melloni RH Jr., Koppel G, Perry KW, Fuller RW, et al (1997) Vasopressin/serotonin interactions in the anterior hypothalamus control aggressive behavior in golden hamsters. J Neurosci 17: 4331–4340. 9151749 10.1523/JNEUROSCI.17-11-04331.1997 PMC6573530

[88] Lyons WE, Mamounas LA, Ricaurte GA, Coppola V, Reid SW, et al (1999) Brain-derived neurotrophic factor-deficient mice develop aggressiveness and hyperphagia in conjunction with brain serotonergic abnormalities. Proceedings of the National Academy of Sciences of the United States of America 96: 15239–15244. 10611369 10.1073/pnas.96.26.15239 PMC24804

[89] Stoltenberg SF, Christ CC, Highland KB (2012) Serotonin system gene polymorphisms are associated with impulsivity in a context dependent manner. Prog Neuropsychopharmacol Biol Psychiatry 39: 182–191. 10.1016/j.pnpbp.2012.06.012 22735397

[90] Ni X, Bismil R, Chan K, Sicard T, Bulgin N, et al (2006) Serotonin 2A receptor gene is associated with personality traits, but not to disorder, in patients with borderline personality disorder. Neurosci Lett 408: 214–219. 17000047 10.1016/j.neulet.2006.09.002

[91] Jakubczyk A, Wrzosek M, Lukaszkiewicz J, Sadowska-Mazuryk J, Matsumoto H, et al (2012) The CC genotype in HTR2A T102C polymorphism is associated with behavioral impulsivity in alcohol-dependent patients. J Psychiatr Res 46: 44–49. 10.1016/j.jpsychires.2011.09.001 21930285 PMC3224206

[92] Saiz PA, Garcia-Portilla P, Paredes B, Corcoran P, Arango C, et al (2011) Role of serotonergic-related systems in suicidal behavior: Data from a case-control association study. Prog Neuropsychopharmacol Biol Psychiatry 35: 1518–1524. 10.1016/j.pnpbp.2011.04.011 21575667

[93] Oades RD, Lasky-Su J, Christiansen H, Faraone SV, Sonuga-Barke EJ, et al (2008) The influence of serotonin- and other genes on impulsive behavioral aggression and cognitive impulsivity in children with attention-deficit/hyperactivity disorder (ADHD): Findings from a family-based association test (FBAT) analysis. Behav Brain Funct 4: 48 10.1186/1744-9081-4-48 18937842 PMC2577091

[94] Wilkie MJ, Smith G, Day RK, Matthews K, Smith D, et al (2009) Polymorphisms in the SLC6A4 and HTR2A genes influence treatment outcome following antidepressant therapy. Pharmacogenomics J 9: 61–70. 10.1038/sj.tpj.6500491 18253134

[95] Rubin DH, Althoff RR, Ehli EA, Davies GE, Rettew DC, et al (2013) Candidate gene associations with withdrawn behavior. J Child Psychol Psychiatry 54: 1337–1345. 10.1111/jcpp.12108 23808549 PMC3800258

[96] Ribases M, Ramos-Quiroga JA, Hervas A, Bosch R, Bielsa A, et al (2009) Exploration of 19 serotoninergic candidate genes in adults and children with attention-deficit/hyperactivity disorder identifies association for 5HT2A, DDC and MAOB. Mol Psychiatry 14: 71–85. 17938636 10.1038/sj.mp.4002100

[97] Ben-Efraim YJ, Wasserman D, Wasserman J, Sokolowski M (2013) Family-based study of HTR2A in suicide attempts: observed gene, gene x environment and parent-of-origin associations. Mol Psychiatry 18: 758–766. 10.1038/mp.2012.86 22751492

[98] Bray NJ, Buckland PR, Hall H, Owen MJ, O'Donovan MC (2004) The serotonin-2A receptor gene locus does not contain common polymorphism affecting mRNA levels in adult brain. Mol Psychiatry 9: 109–114. 14699448 10.1038/sj.mp.4001366

[99] Kusumi I, Suzuki K, Sasaki Y, Kameda K, Sasaki T, et al (2002) Serotonin 5-HT(2A) receptor gene polymorphism, 5-HT(2A) receptor function and personality traits in healthy subjects: a negative study. J Affect Disord 68: 235–241. 12063151 10.1016/s0165-0327(00)00324-4

[100] Spurlock G, Heils A, Holmans P, Williams J, D'Souza UM, et al (1998) A family based association study of T102C polymorphism in 5HT2A and schizophrenia plus identification of new polymorphisms in the promoter. Mol Psychiatry 3: 42–49. 9491812 10.1038/sj.mp.4000342

[101] Erdmann J, Shimron-Abarbanell D, Rietschel M, Albus M, Maier W, et al (1996) Systematic screening for mutations in the human serotonin-2A (5-HT2A) receptor gene: identification of two naturally occurring receptor variants and association analysis in schizophrenia. Hum Genet 97: 614–619. 8655141 10.1007/BF02281871

[102] Ozaki N, Manji H, Lubierman V, Lu SJ, Lappalainen J, et al (1997) A naturally occurring amino acid substitution of the human serotonin 5-HT2A receptor influences amplitude and timing of intracellular calcium mobilization. J Neurochem 68: 2186–2193. 9109547 10.1046/j.1471-4159.1997.68052186.x

[103] Hazelwood LA, Sanders-Bush E (2004) His452Tyr polymorphism in the human 5-HT2A receptor destabilizes the signaling conformation. Mol Pharmacol 66: 1293–1300. 15496511

[104] Blasi G, De Virgilio C, Papazacharias A, Taurisano P, Gelao B, et al (2013) Converging evidence for the association of functional genetic variation in the serotonin receptor 2a gene with prefrontal function and olanzapine treatment. JAMA Psychiatry 70: 921–930. 10.1001/jamapsychiatry.2013.1378 23842608

[105] Griffiths-Jones S (2006) miRBase: the microRNA sequence database. Methods Mol Biol 342: 129–138. 16957372 10.1385/1-59745-123-1:129

[106] Griffiths-Jones S, Saini HK, van Dongen S, Enright AJ (2008) miRBase: tools for microRNA genomics. Nucleic Acids Res 36: D154–158. 17991681 10.1093/nar/gkm952 PMC2238936

[107] Kovacs-Nagy R, Elek Z, Szekely A, Nanasi T, Sasvari-Szekely M, et al (2013) Association of aggression with a novel microRNA binding site polymorphism in the wolframin gene. Am J Med Genet B Neuropsychiatr Genet 162B: 404–412. 10.1002/ajmg.b.32157 23650218

[108] Meng Y, Shao C, Ma X, Wang H (2013) Introns targeted by plant microRNAs: a possible novel mechanism of gene regulation. Rice (N Y) 6: 8. 24280590 10.1186/1939-8433-6-8 PMC4883735

[109] Westra H-J, Peters MJ, Esko T, Yaghootkar H, Schurmann C, et al (2013) Systematic identification of trans eQTLs as putative drivers of known disease associations. Nat Genet 45: 1238–1243. 10.1038/ng.2756 24013639 PMC3991562

[110] Harms N, Ras J, Reijnders WN, van Spanning RJ, Stouthamer AH (1996) S-formylglutathione hydrolase of Paracoccus denitrificans is homologous to human esterase D: a universal pathway for formaldehyde detoxification? J Bacteriol 178: 6296–6299. 8892832 10.1128/jb.178.21.6296-6299.1996 PMC178503

[111] Wu D, Li Y, Song G, Zhang D, Shaw N, et al (2009) Crystal structure of human esterase D: a potential genetic marker of retinoblastoma. FASEB J 23: 1441–1446. 10.1096/fj.08-125286 19126594

[112] Thierry-Mieg D, Thierry-Mieg J (2006) AceView: a comprehensive cDNA-supported gene and transcripts annotation. Genome Biol 7 Suppl 1: S12 11–14. 16925834 10.1186/gb-2006-7-s1-s12 PMC1810549

[113] Liu X, Yu X, Zack DJ, Zhu H, Qian J (2008) TiGER: a database for tissue-specific gene expression and regulation. BMC Bioinformatics 9: 271 10.1186/1471-2105-9-271 18541026 PMC2438328

[114] Chiao JY, Blizinsky KD (2010) Culture-gene coevolution of individualism-collectivism and the serotonin transporter gene. Proc Biol Sci 277: 529–537. 10.1098/rspb.2009.1650 19864286 PMC2842692

[115] Miller GA (2010) Mistreating Psychology in the Decades of the Brain. Perspect Psychol Sci 5: 716–743. 21949539 10.1177/1745691610388774 PMC3177535

